# Oxidative stress biomarkers in dogs with benign prostatic hyperplasia

**DOI:** 10.1186/s13620-022-00228-3

**Published:** 2022-12-15

**Authors:** Anna Domoslawska, Sławomir Zduńczyk, Marta Kankofer, Arleta Bielecka

**Affiliations:** 1 Department of Animal Reproduction with Clinic, University of Warmia and Mazury, Oczapowskiego 14, 10-719 Olsztyn, Poland; 2 Department of Biochemistry, Faculty of Veterinary Medicine, University of Life Sciences, Akademicka 12, 20-033 Lublin, Poland

**Keywords:** Benign prostatic hyperplasia, Dogs, Oxidative stress

## Abstract

**Background:**

The aim of this study was to evaluate total antioxidant capacity (TAC) and biomarkers of lipid and protein peroxidation in the blood serum of dogs with benign prostatic hyperplasia (BPH). The study was conducted on 36 intact male dogs of various breeds. The dogs were assigned to two groups: BPH group (*n* = 18) and non-affected group (*n* = 18). Blood samples were collected from the cephalic vein. The antioxidant status of the serum was assessed using TAC. The levels of bityrosine, formylkynurenine and SH-groups were used as protein peroxidation biomarkers and the level of radical cations of N,N-diethyl-paraphenylene diamine (RC-DEPPD) was used as a marker for lipid peroxidation. TAC and the concentrations of SH-groups and RC-DEPPD in the serum were determined spectrophotometrically, the concentrations of bityrosine and formylokynurenine, were determined using spectrofluorimetric methods.

**Results:**

The mean value of TAC in the serum was significantly lower (*P* = 0.01) in BPH dogs than in non-affected dogs (3.10 ± 0.56 vs 4.20 ± 1.60 μmol/g protein). Mean levels of protein and lipid oxidation biomarkers showed a trend towards oxidative imbalance, but there were no statistically significant differences between dogs with BPH and controls (*P* > 0.05).

**Conclusion:**

In conclusion, significantly lower serum TAC in dogs with BPH compared to non-affected dogs suggests a potential involvement of oxidative stress in the pathogenesis of BPH in dogs. More studies are needed to clarify the role of oxidative stress in the development of BPH in dogs.

## Background

Benign prostatic hyperplasia (BPH) is considered the most common prostatic disease in dogs. Around 80% of sexually intact dogs older than 5 years and 95% dogs older than 9 years show gross or microscopic changes related to BPH [1]. BPH is one of the major causes of infertility in male dogs [2–4]. The pathogenesis of BPH is still not fully understood. It is assumed that BPH develops under the influence of dihydrotestosterone (DHT), an active metabolite of testosterone [5–8]. With age, estrogen concentration increases and testosterone concentration decreases. The altered ratio of estrogen to testosterone leads to an increased concentration of androgen receptor and an increased conversion of testosterone to DHT in the prostate, which leads to the proliferation (hyperplasia) and enlargement (hypertrophy) of glandular cells [9].

Several studies showed that oxidative stress may also play a role in the development of BPH in men [10–13]. Oxidative stress is an imbalance between the production of reactive oxygen species (ROS) and the ability of the antioxidant mechanism to neutralize these reactive products. Recently, it has been presumed that age-related hormonal changes or infection activate a chronic inflammatory response in the prostate [14–16]. This chronic prostatic inflammation causes the generation of free radicals and results in oxidative stress. This leads to oxidative DNA damage and hyperplastic transformation of prostatic cells [13, 17, 18]. Thus, oxidative stress is not just a phenomenon accompanying BPH, but plays a role in its pathogenesis.

There are very few studies on oxidative stress in dogs with BPH, and these have conflicting results. For instance, Dearakhshandeh et al. (2019) [19] reported a decrease in antioxidant enzyme level activities in the blood serum of dogs induced BPH by testosterone and estradiol. However, Angrimani et al. (2020) [20] found no significant differences in the oxidative profile of prostatic fluid between BPH dogs and non-affected dogs.

Antioxidant status can be assessed by the determination of total antioxidant capacity (TAC) and oxidative stress by the estimation of end products of peroxidation. TAC can indicate the antioxidant response against the free radicals in a biological sample [21, 22]. A high level of ROS may result in protein peroxidation, leading to the production of amino acids carbonyl derivatives, bityrosine and formylkynurenine and to a decrease in sulfhydryl-groups (SH-groups) content [23]. As a marker for lipid peroxidation, radical cations of N,N-diethyl-paraphenylene diamine (RC-DEPPD) can be used [24, 25].

TAC as well as lipid and protein peroxidation in the blood serum of dogs with BPH have not been investigated until now. There are only isolated reports of indicators of lipid peroxidation in dogs with BPH [19, 20]. Thus, the aim of this study was to evaluate TAC and biomarkers of lipid and protein peroxidation in the blood serum of dogs with BPH.

## Results

The age of the dogs ranged from 5 to 8 years and averaged 7.1 ± 1.3 years in the BPH group and 6.5 ± 1.2 years in the control group. Animals in both groups were of similar weight.

The mean values of TAC, bityrosine, formylkynurenine, SH-groups and RC-DEPPD in the serum of BPH and non-affected dogs are presented in Table 1. The mean value of the TAC in the serum was significantly lower (*P* = 0.01) in BPH dogs than in non-affected dogs (3.10 ± 0.56 vs 4.20 ± 1.60 μmol/g protein). The mean concentrations of protein and lipid peroxidation biomarkers in serum were not statistically different between dogs with BPH and controls. However, there was an increasing tendency in mean concentrations of bityrosine (*P* = 0.07), formylkynurenine and RC-DEPPD (*P* = 0.06) in dogs with BPH compared to non-affected dogs.

**Table 1:** Values of total antioxidant capacity (TAC), bityrosine (BT), formylkynurenine (FK), SH-groups and RC-DEPPD (mean ± SEM) in serum of BPH and non-affected dogs

Parameter	BPH	Control	*P* value
TAC μmol/g protein	3.10 ± 0.41^a^	4.20 ± 0.19^b^	0.01
BT μg/mg protein	0.45 ± 0,02	0.40 ± 0.02	0.07
SH μmol/g protein	3.26 ± 0.11	3.89 ± 0.18	0.56
FK μg/mg protein	0.16 ± 0.01	0.15 ± 0.01	0.13
RC-DEPPD mmol/g protein	0.06 ± 0.003	0.05 ± 0.001	0.06

^a, b^difference statistically significant at *P* < 0.05

## Discussion

Our study showed that serum TAC was significantly lower in dogs with BPH than in non-affected dogs. This indicates the possible importance of reduced antioxidant defense in the development of BPH in dogs. So far there are no data available about the serum TAC of dogs with spontaneous BPH. Only Dearakhshandeh et al. (2019) [19] reported a significant decrease in the activity of antioxidant enzymes glutathione peroxidase and superoxide dismutase in the serum of dogs with BPH induced by testosterone and estradiol, compared with control dogs. In comparison, decreased efficiency of antioxidant mechanisms has been reported in several studies on men with BPH [10–13].

Human and canine prostate glands share many functional, anatomical and physiological features and BPH is one of the most important problems in both intact male dogs and men [26, 27]. Oxidative stress have been considered to play a role in the development of BPH in men [10–13]. It is hypothesised that BPH is an immune-mediated inflammatory disease [14–16]. Chronic inflammatory response in the prostate following changes in sex steroid hormones or infection leads to accumulation of immunocompetent cells in the prostate, such as macrophages and neutrophils, which generate reactive oxygen and nitrogen species. Increased production of free radicals can deplete the antioxidant defense system and result in a decrease in TAC and oxidative stress. Oxidative damage of prostatic tissue leads to compensatory cellular proliferation with resulting hyperplastic growth [11, 17, 18].

Oxidative stress can be increased by antioxidant deficiency. Reduced concentration of antioxidants such as vitamin E and C, beta-carotene, ascorbate and selenium were found in men with BPH compared to control, and the use of antioxidants in the prevention and treatment of BPH is under discussion [28–31]. Our previous study showed suboptimal Se and vitamin E status in male dogs with lowered fertility [32].

Bityrosine, formylkynurenine and SH-groups were determined as biomarkers of protein peroxidation. Serum levels of bityrosine and formylkynurenine were numerically higher and SH-groups were numerically lower in dogs with PBP compared to controls, but the differences were not statistically significant. Protein peroxidation intensity in the serum of dogs with BPH has not been investigated until now. However, studies in men with BPH showed elevated serum levels of carbonylated proteins, a marker of protein peroxidation [33, 34].

The results of the present study did not show a significant increase in the RC-DEPPD, a marker of lipid peroxidation. There are only very few studies on lipid peroxidation in dogs with BPH. Similarly to our study, Dearakhshandeh et al. (2019) [19] found no significant differences in serum levels of malonodialdehyde (MDA), one of the products of lipid peroxidation, between dogs with induced BPH and controls. Angrimani et al. (2020) [20] reported no significant increase in thiobarbituric acid reactive substances (TBARS), another marker of lipid peroxidation, in the prostatic fluid of dogs with BPH compared to controls. However, some studies showed increased lipid peroxidation in men with BPH compared to controls [10, 11, 33].

Thus, the results of our study only indicate a trend towards oxidative imbalance in the systemic plasma of dogs with BPH. However, oxidation stress may be local. Pace et al. (2010) [12] reported that in men with BPH, the increase in plasma peroxides was more pronounced in samples taken locally during prostatectomy than taken systemically. Bergsma et al. (2022) [35] found no associations between oxidation stress biomarker concentrations in systemic plasma and seminal plasma in infertile men. This may indicate that systemic oxidation stress and local oxidation stress are not strongly interrelated and support the notion that the male genital tract may have a local redox-controlled microenvironment that is responsible for the overall redox balance.

We are aware that our study also has limitations. BPH was diagnosed only on the basis of ultrasound examination and clinical symptoms. Ultrasonography is the method of choice for the presumptive diagnosis of BPH in dogs, but sometimes this underdiagnoses the early stages of clinical BPH [36, 37]. Clinical signs of BPH are non-specific and similar to those of other prostate diseases [7, 37]. Prostatic biopsy for cytology or histology is considered the gold standard for the diagnosis of BPH in the living animal, but is invasive and therefore uncommon [7, 38]. An alternative for confirmation of BPH is analysis of serum concentration of the biomarker canine prostate specific esterase (CPSE) biomarker. Serum CPSE concentrations are significantly higher in dogs with BPH than in normal dogs [39–41]. Our study was conducted on a clinical population of client-owned dogs and the dog owners did not consent to the biopsy. The entire serum obtained was used for determination of oxidative stress biomarkers and CPSE determination was not performed.

## Conclusion

In conclusion, our study showed significantly lower TAC and an increasing trend in biomarkers of protein and lipid peroxidation in the serum of BPH dogs compared to control dogs. This suggests a potential involvement of oxidative stress in the pathogenesis of BPH in dogs. More studies are needed to clarify the role of oxidative stress in the pathogenesis of BPH in dogs.

## Material and methods

### Animals and study design

The study was conducted on 36 intact stud dogs of various breeds. The males were presented because of a sanguineous discharge from the urethra or for the evaluation of semen quality. The dogs were assigned to two groups: a BPH group (*n* = 18) and a non-affected group (*n* = 18). The diagnosis of BPH was based on history, clinical symptoms such as sanguineous discharge from the urethra, dysuria, tenesmus and enlargement of the prostate on rectal palpation and ultrasound examination (Mindray Bio-Medical 2 with a 7.5 MHz convex transducer). The control animals showed no clinical signs and the prostate was not enlarged. The dogs were fed with commercial premium dry diets. The study was conducted according to good veterinary practice and had informed owner consent for the study.

### Blood collection

Blood samples were collected from the cephalic vein into plastic tubes and samples were left for clotting at room temperature for 20 min. The serum was obtained after centrifugation at 3000 g for 5 min at room temperature. Samples were stored frozen at − 20 °C until further use.

### TAC determination

TAC was measured according to the method of Benzie and Strain (1996) [42], which is based on the ferric-reducing ability of the samples. A working reagent consisting of 300 mmol/dm^3^ acetate buffer (pH 3.6), 10 mmol/dm^3^ 2,4,6-tri-pyridyl-s-triazine (TPTZ, Sigma, Poznań, Poland) in 40 mmol/dm^3^ HCl and 20 mmol/dm^3^ FeCl_3_ x 6H_2_O mixed at a ratio of 10:1:1, was prepared immediately before use. The working reagent (2250 μL) was mixed with 25 μl of sample and absorbance was measured at 593 nm (Ultrospec 2000, Pharmacia, Sweden) against the working reagent alone. After exactly 10 min of incubation at room temperature, the absorbance was read again. The difference in absorbance at zero and 10 min time was compared with the standard curve prepared with different dilutions of Fe (II) between 0 and 1000 μmol/dm^3^. The results were expressed in μmol per g protein in the sample.

### Bityrosine determination

Bityrosine was determined using a spectrofluorimetric method according to Rice-Evans et al. (1991) [23]. Samples were diluted with the 0.9% NaCl, the fluorescence was measured at an excitation of 325 nm and emission 410 nm. The spectrofluorimeter (Jasco, Tokyo, Japan) was standardized as described above. The results were expressed as μg/mg protein. The intra-assay and inter-assay coefficients of variation were 5.8% (*n* = 10) and 5.9% (*n* = 10), respectively.

### Formylkynurenine determination

Formylkynurenine was determined using a spectrofluorimetric method according to Rice-Evans et al. (1991) [23]. After a previous 50-fold dilution of the samples with the 0.9% NaCl, the fluorescence was measured at an excitation of 360 nm and emission 454 nm. The spectrofluorimeter (Jasco, Tokyo, Japan) was standardized to 100 deflections with chinine sulphate (0.1 μg/mL in 0.1 mol/ H2SO4) at an excitation of 350 nm and emission 445 nm. The results were expressed as μg/mg protein. The intra-assay and inter-assay coefficients of variation were 6.1% (*n* = 10) and 6.4% (*n* = 10), respectively.

### SH-groups determination

The content of SH-groups was determined as described by Rice-Evans et al. (1991) [23]. A volume of 300 μL of 10% sodium dodecyl sulphate (SDS, Sigma, Poznań, Poland) in sodium phosphate buffer (10 mmol/dm^3^, pH 8.0) was added to 300 μL of sample and mixed thoroughly. A 2.4 mL aliquot of the same buffer was added and absorbance was measured at 412 nm (Ultrospec 2000, Pharmacia, Sweden). After measurement, 300 μL of DTNB (20 mg of 5,5′-dithiobis-2-nitro benzoate, Sigma, in 50 ml of buffer) was added and incubated for 1 hour at 37 °C. The control contained 300 μL of buffer instead of DTNB. After incubation, absorbance was measured again at 412 nm. The difference in absorbance before and after incubation (after subtraction of adequate absorbance of control) referred to the content of the SH-groups. The content was calculated using a standard curve prepared with different dilutions of glutathione (GSH, Sigma, 0-1 mmol/dm^3^ in buffer) and expressed in μmol per g of protein. The intra-assay and inter-assay coefficients of variation were 6.9% (*n* = 10) and 7.1% (*n* = 10), respectively.

### RC-DEPPD determination

The concentration of RC-DEPPD was determined as described by Alberti et al. (2000) [24]. The estimation of RC-DEPPD formed in the reaction of alkoxy and peroxy radicals derived from hydroperoxides (present in the sample) was performed using an indirect estimation of the level of hydroperoxides. The incubation mixture contained 1 ml of acetate buffer (pH 4.8), 10 μL of an aqueous solution of DEPPD (Sigma, Poznan, Poland) (0.37 mol/l) and 20 μL of plasma. After 1.5 h incubation at 37^o^ C absorbance was read at 505 nm against distilled water (Ultrospec 2000, Pharmacia, Uppsala, Sweden). In the control sample, 20 μL of distilled water replaced the plasma. Calculations were based on a standard curve prepared with six different dilutions of H_2_O_2_. The results were expressed as mmol/g protein.

### Protein determination

Protein concentration in the samples was determined according to the method based on the biuret reaction using a commercial colorimetric kit (Cormay, Lublin, Poland).

### Statistical analysis

The results were presented as mean and standard error of median (SEM) and compared between both groups using a Mann-Whitney or t-Student test (GraphPAD PRISM, Version 9.3.1, GraphPad Software, San Diego, CA, USA).

## Data Availability

All data generated or analysed during this study are included in this published article.
